# An anthropometric study of sexual orientation and gender identity in Thailand

**DOI:** 10.1038/s41598-021-97845-9

**Published:** 2021-09-16

**Authors:** Malvina N. Skorska, Lindsay A. Coome, Diana E. Peragine, Madison Aitken, Doug P. VanderLaan

**Affiliations:** 1 Child & Youth Psychiatry, Centre for Addiction and Mental Health, Toronto, ON M6J 1H4 Canada; 2 Department of Psychology, University of Toronto Mississauga, 3359 Mississauga Rd. N., Mississauga, ON L5L 1C6 Canada; 3 Department of Psychiatry, University of Toronto, Toronto, ON M5T 1R8 Canada

**Keywords:** Psychology, Human behaviour

## Abstract

The biodevelopment of psychological sex differentiation is putatively reflected in several anthropometrics. We examined eight anthropometrics in 1404 Thai participants varying in sex, sexual orientation, and gender identity/expression: heterosexual men and women, gay men, lesbian women, bisexual women, *sao praphet song* (transgender birth-assigned males), *toms* (transgender birth-assigned females), and *dees* (birth-assigned females attracted to *toms*). Exploratory factor analyses indicated the biomarkers should be analyzed independently. Using regressions, in birth-assigned males, less male-typical second-to-fourth digit ratios in the left hand were associated with sexual orientation towards men regardless of gender identity/expression, whereas shorter height and long-bone growth in the arms and legs were more evident among *sao praphet song*—who are both sexually oriented towards men and markedly feminine. In birth-assigned females, there were no clear sexual orientation effects, but there were possible gender-related effects. Groups of individuals who tend to be more masculine (i.e., *toms*, lesbians) showed more male-typical patterns on weight and leg length than some groups of individuals who tend to be less masculine (i.e., heterosexual women, *dees*). Thus, it appears the various anthropometrics inform separate biodevelopmental processes that differentially relate to sexual orientation and gender identity/expression depending on the measure in question as well as birth-assigned sex.

## Introduction

Sex differences in the body, brain, and behavior are among the largest dimensions of variation in humans^1^. The precise roles of biological mechanisms in the development of sex differences are as yet not entirely clear. Neurohormonal theory argues that sex differentiation of the brain and body is largely due to the influence of gonadal hormones (e.g., testosterone) during pre- and perinatal development^2,3^, although genetic and immunological factors likely also play a role^4–6^. Brain development occurs in parallel with the development of other physical characteristics (e.g., genital formation^7^). Variations in testosterone exposure may thus leave a “fingerprint” on the body along with shaping the brain and subsequent behavior. As a result, physical markers of putative biological processes, or “biomarkers,” might help inform our understanding of the process of psychological sexual differentiation^4^.

In this article, “sex” is used to refer to readily observable somatic characteristics (e.g., genitals). “Male” and “female” are used in reference to sex-assigned-at-birth and are not intended to refer to the sociocultural construct of gender. When referring to a specific group of individuals who are not cisgender (i.e., birth-assigned sex and gender identity do not align relative to cultural norms), the culturally relevant identity terms are used.

To inform psychological sex differentiation, one can examine how biomarkers relate to psychological domains that are differentiated by sex. Sexual orientation and gender expression (i.e., gender identity; masculine or feminine gender-typed behavior) are among the largest psychological sex differences^8–10^. Within-sex variation in these traits provides useful human models for examining sex differentiation of the brain and the development of these traits. The majority of individuals are sexually attracted to individuals of the other birth sex and display a gender identity/expression that corresponds with their birth sex. Individuals who are sexually oriented toward individuals of the same birth sex and/or display gender identity/expression that is most commonly seen among members of the other birth sex may have experienced biological processes that are more aligned with the other birth sex. If so, biomarkers of these processes should evidence within-sex variation that corresponds to sexual orientation and gender expression.

Several biomarkers have been identified and have often been studied in isolation from one another. The ratio of the length of the index finger to the length of the ring finger (i.e., 2D:4D or digit ratio) is thought to be influenced by prenatal androgen exposure, with females tending to have a higher ratio than males^10,11^. Some studies reported a female-typical (i.e., higher) digit ratio in gay men, but a meta-analysis found no association between sexual orientation and digit ratios in males, whereas in females, lesbians exhibited a more male-typical digit ratio compared with heterosexuals^2,10,12^. Few studies have been conducted on transgender individuals and there are mixed results across studies. Some studies suggest digit ratios were more consistent with gender identity/expression than with birth-assigned sex^13–15^. A recent study and meta-analysis indicated no difference in 2D:4D between trans men and cisgender women, whereas trans women had a more female-typical digit ratio compared with cisgender men^16^.

Height is another sexually differentiated human trait that has genetic, prenatal hormonal, and psychosocial (e.g., nutrition, stress) influences^17–19^. Most studies found no height difference between lesbian and heterosexual women^20–22^, cf.^23^; however, several studies indicated that same-sex attracted males were shorter than other-sex attracted males^20–22,24^. There is also some evidence that same-sex attracted birth-assigned males who experience gender dysphoria were shorter than same-sex attracted males who did not experience gender dysphoria^25^.

Greater skeletal size in males, compared with females, becomes apparent in childhood, accelerates in adolescence^26,27^, and is especially pronounced in the long bones of the legs and arms^28^. Long bone growth is responsive to hormonal signals throughout development, primarily androgens^29^. The one study of sexuality and long bone length found adult gay men had shorter long bones in the legs and arms compared with adult heterosexual men, and lesbian women had longer long bones in the legs and arms than heterosexual women^30^.

Hand bones are also influenced by gonadal hormones during development^31–33^. The ratio of hand width-to-length reveals a sex difference such that heterosexual men’s hands were relatively wider than those of heterosexual women (i.e., a larger ratio in men^30^). In the one report to date, heterosexual men and lesbian women had larger hand width-to-length ratios than gay men and heterosexual women, respectively^30^.

Weight is also sexually differentiated, with males being heavier on average than females^34^, even when accounting for height^35^. Testosterone is thought to play a direct role in the regulation of weight and adipose tissue distribution, and androgen receptors can be found in human adipose tissue^34^, although weight is also influenced by social factors^36^. Across studies, lesbian women were heavier than heterosexual women^23^, and both cisgender and transgender same-sex attracted birth-assigned males were lighter than heterosexual men^24,25,30^.

An unanswered question is whether these various biomarkers provide windows into the same or different developmental events. Some biomarkers have, for example, exhibited similar patterns when examined separately in either sexual orientation or gender identity groups (e.g., height and weight for sexual orientation in birth-assigned males), suggesting they might serve as proxies of the same developmental processes. An alternate possibility is that different biomarkers are indicative of different processes, with little-to-no overlap (e.g., height vs. 2D:4D findings for sexual orientation in birth-assigned males). To evaluate these possibilities, we used exploratory factor analysis (EFA) to examine covariation among the anthropometrics reviewed above. We did so in the entire sample and also considered measurement invariance across varying sexual orientation and gender identity/expression groups to test whether a similar factor structure applied to each group. If a similar factor structure was present, it would suggest that common latent biological processes underlie the factors identified. To this end, we examined a comprehensive—and, arguably, the most complete—set of biomarkers considered in this literature to date.

Importantly, there is some research suggesting that 2D:4D is related to physical size^37,38^. If sex differences in 2D:4D were the result of allometry rather than of biological mechanisms (e.g., prenatal androgens) hypothesized to have more targeted developmental effects on the phalanges, it would suggest that investigating digit ratio as a biomarker is of limited utility. We, therefore, tested for allometry in 2D:4D. Specifically, regression analyses examined whether digit ratio was predicted by sex, physical size, or their interaction. If the sex difference in 2D:4D reflects allometry, then more male-typical (lower) digit ratios should be associated with larger physical size^39^.

We also investigated the unanswered question of whether the biological mechanisms underlying anthropometric biomarkers have a stronger influence in groups where sexual orientation and gender identity are both similar to patterns that are most commonly found among the other sex. For example, male same-sex sexual attraction was associated more strongly with a putative biomarker of immune factors influencing sexual orientation—numbers of older brothers (i.e., the fraternal birth order effect)—in more feminine/transgender, compared with more masculine/cisgender, individuals^40,41^. In most previous studies, however, sexual orientation and gender expression have been challenging to disentangle because they overlap to some extent. Same-sex attracted individuals display greater gender nonconformity compared with other-sex attracted individuals^42^, and transgender individuals display higher rates of sexual attraction to the same birth-assigned sex compared with individuals in the general population^43^.

To begin to address this gap, our study was conducted in Thailand, where sexual orientation and gender diversity are more visible and socially tolerated than in Western culture^44,45^. In addition to binary categories of “male/boy/man” and “female/girl/woman,” Thai culture recognizes several distinct “third” or nonbinary gender categories^45,46^. Birth-assigned males who adopt a feminine gender identity/role are known as *sao praphet song* (translated as a “second kind of woman”) and are primarily androphilic (i.e., sexually attracted to males)^45,47^. Birth-assigned females who adopt a masculine gender identity/role are known locally as *toms*, derived from the English word “tomboy”; *toms* are primarily gynephilic (i.e., sexually attracted to females)^45^. Females with a feminine gender expression and who engage in sexual and/or romantic relationships with *toms* are locally known as *dee*s, derived from the latter syllable of the English word “lady”^45^. Thai gay men, lesbian women, and bisexual individuals occupy roles similar to their counterparts in the West^45–47^.

Thus, the present study examines a comprehensive set of biomarkers (i.e., 2D:4D, height, leg length, arm length, hand width-to-length ratio, weight) in a large and diverse Thai sample in both sexes. This approach is uniquely suited to identify associations (or lack thereof) between biomarkers, while also addressing whether differences in biomarker expression patterns are similarly or differentially related to within-sex variation in sexual orientation and gender identity/expression. We predicted within-sex differences would be greater between heterosexual participants and participants from groups where both gender identity/expression and sexual orientation vary relative to birth-assigned sex, compared to groups where only sexual orientation varies (e.g., lesbian women). Specifically, *sao praphet song* and *toms* are more markedly similar to heterosexuals of the other birth-assigned sex in their gender identity/expression than individuals who identify as gay, lesbian, bisexual, or *dee*—although all of these groups exhibit attraction to the same birth-assigned sex^45–48^. Thus, we expected *sao praphet song* to differ the most from heterosexual men on biomarkers, and *toms* to differ the most from heterosexual women. We also expected that gay men would differ, although to a lesser extent, from heterosexual men, and lesbian women, bisexual women, and *dees* would differ, to a lesser extent from heterosexual women. Notably, by employing a Thai sample, we are also uniquely poised to test cross-cultural (in)consistencies in biomarker expression. If the various biomarkers are proxies for sex-differentiated processes that influence nervous system development, we should replicate sex, sexual orientation, and gender identity/expression differences found in other populations.

## Results

### Exploratory factor analysis (EFA)

Using Mplus, the EFA in the full sample revealed three factors and fit the data reasonably well; however, when examining the factor structure within each group, we did not retrieve the same or reasonably similar factor structure. Thus, we could not demonstrate this initial aspect of measurement invariance, suggesting that the factor structure did not hold across groups (details are in the “Supporting Information”). We therefore investigated allometry in 2D:4D and then each biomarker individually.

### Allometry in 2D:4D

Table 1 summarizes the results of each of the allometry-related regressions (see “Methods” for descriptions). Table S13 in the “Supporting Information” includes the descriptive statistics for the physical size variables by group. In regressions 1–4 and 6, there were significant effects of sex (Cohen *d*s range from − 0.23 to − 0.59) and non-significant effects of average finger length (*d*s range from less than 0.01–0.02), supportive of isometry. In regression 5, there was a significant effect of sex (*d* = − 0.54) and a small positive significant effect of average right-hand finger length (*d* = 0.03), which is in the opposite direction of what would be expected if lower 2D:4D was associated with greater physical size.

**Table 1:** Results of regressions testing for allometry in relation to 2D:4D.

	DV	Main effect variables	*n*	Main effect statistics^a^		*d*	95% CI for *d*		Interaction statistics^b^		*d*	95% CI for *d*	
				*β*	*p*				*Β*	*p*			
1	Average 2D:4D	**Het women vs het men** Average finger length	552	− 0.570 0.102	**< 0.001** 0.061	− 0.59 0.02	− 0.77 − 0.15	− 0.42 0.19	− 0.662	0.433	− 0.02	− 0.18	0.15
2	Average 2D:4D	**AFAB vs AMAB** Average finger length	1304	− 0.342 0.019	**< 0.001** 0.584	− 0.35 < 0.01	− 0.46 − 0.010	− 0.24 0.11	− 0.608	0.260	− 0.02	− 0.13	0.09
3	Left 2D:4D	**Het women vs het men** Average left finger length	554	− 0.492 0.083	**< 0.001** 0.168	− 0.51 0.02	− 0.68 − 0.15	− 0.34 0.18	0.579	0.535	0.02	− 0.15	0.18
4	Left 2D:4D	**AFAB vs AMAB** Average left finger length	1307	− 0.224 − 0.012	**0.002** 0.747	− 0.23 < 0.01	− 0.34 − 0.11	− 0.12 0.11	0.113	0.847	< 0.01	− 0.11	0.11
5	Right 2D:4D	**Het women vs het men** **Average right finger length**	554	− 0.518 0.119	**< 0.001** **0.023**	− 0.54 0.03	− 0.71 − 0.14	− 0.37 0.19	− 1.241	0.124	− 0.03	− 0.20	0.13
6	Right 2D:4D	**AFAB vs AMAB** Average right finger length	1306	− 0.365 0.045	**< 0.001** 0.174	− 0.37 0.01	− 0.48 − 0.10	− 0.26 0.12	− 0.995	0.053	− 0.03	− 0.14	0.08
7	Average 2D:4D	**Het women vs het men** Average hand length	552	− 0.611 0.114	**< 0.001** 0.061	− 0.64 0.01	− 0.81 − 0.16	− 0.47 0.18	− 0.673	0.513	− 0.01	− 0.17	0.16
8	Average 2D:4D	**AFAB vs AMAB** Average hand length	1303	0− .382 0.045	**< 0.001** 0.249	− 0.39 < 0.01	− 0.50 − 0.10	− 0.28 0.11	− 0.776	0.242	− 0.01	− 0.12	0.10
9	Left 2D:4D	**Het women vs het men** Left hand length	554	− 0.507 0.081	**< 0.001** 0.164	− 0.53 0.01	− 0.69 − 0.16	− 0.36 0.17	0.372	0.699	< 0.01	− 0.16	0.17
10	Left 2D:4D	**AFAB vs AMAB** Left hand length	1306	− 0.122 0.000	**0.002** 0.904	− 0.25 < 0.01	− 0.36 − 0.11	− 0.14 0.11	0.090	0.889	< 0.01	− 0.11	0.11
11	Right 2D:4D	**Het women vs het men** Right hand length	554	− 0.542 0.116	**< 0.001** 0.061	− 0.56 0.01	− 0.73 − 0.16	− 0.39 0.18	− 1.582	0.127	− 0.02	− 0.18	0.15
12	Right 2D:4D	**AFAB vs AMAB** Right hand length	1305	− 0.204 0.006	**< 0.001** 0.074	− 0.42 0.01	− 0.53 − 0.10	− 0.31 0.11	− 1.429	**0.028**	− 0.02	− 0.12	0.09
13	Average 2D:4D	**Het women vs het men** Height	548	− 0.619 0.118	**< 0.001** 0.097	− 0.65 0.01	− 0.82 − 0.15	− 0.48 0.18	− 2.247	0.073	− 0.03	− 0.19	0.14
14	Average 2D:4D	**AFAB vs AMAB** **Height**	1276	− 0.456 0.094	**< 0.001** **0.034**	− 0.47 0.01	− 0.58 − 0.10	− 0.36 0.12	− 1.623	0.058	− 0.02	− 0.13	0.09
15	Left 2D:4D	**Het women vs het men** Height	550	− 0.480 0.061	**< 0.001** 0.368	− 0.50 0.01	− 0.67 − 0.16	− 0.33 0.17	− 0.990	0.421	− 0.01	− 0.18	0.16
16	Left 2D:4D	**AFAB vs AMAB** Height	1279	− 0.307 0.048	**< 0.001** 0.269	− 0.31 0.01	− 0.42 − 0.10	− 0.20 0.12	− 0.709	0.399	− 0.01	− 0.12	0.10
17	Right 2D:4D	**Het women vs het men** Height	550	− 0.574 0.134	**< 0.001** 0.057	− 0.60 0.02	− 0.77 − 0.15	− 0.43 0.18	− 2.858	**0.021**	− 0.03	− 0.20	0.13
18	Right 2D:4D	**AFAB vs AMAB** **Height**	1278	− 0.475 0.111	**< 0.001** **0.011**	− 0.49 0.01	− 0.60 − 0.10	− 0.38 0.12	− 2.112	**0.012**	− 0.03	− 0.03	0.08

Bold, p < .05

A first set of regressions^a^ only included main effects of sex and finger length, hand length, or height and a second set of regressions^b^ included main effects and the interaction between sex and finger length, hand length, or height. Age and experimenter were also included in regressions to control for these variables (see “Supporting Information” for more details). Heterosexual women (coded 0) versus heterosexual men (coded 1); individuals assigned female at birth (AFAB) (i.e., heterosexual women, lesbian women, bisexual women, *tom*s, *dee*s) (coded 0) versus individuals assigned male at birth (AMAB) (i.e., heterosexual men, gay men, *sao praphet song*) (coded 1). The “Supporting Information” indicates how *d*s were calculated. Regressions were conducted in Mplus.

*Het* heterosexual, *CI* confidence interval.

Allometry could also be demonstrated using hand length and height, given these are indices of physical size. Thus, regressions 7–12 had average 2D:4D, left 2D:4D, or right 2D:4D as the dependent variable and average hand length, left hand length, or right hand length as the independent variable that provided an index of physical size. In all regressions, there were significant effects of sex (*d*s range from − 0.25 to − 0.64) and not of hand length (*d*s ≤ 0.01). In regression 12, there was a significant interaction between the AFAB versus AMAB variable and right hand length, such that the relationship between right 2D:4D and right hand length was not significant among AMAB participants (*β* = − 0.001, *p* = 0.764, *n* = 633) but positive and significant among AFAB participants (*β* = 0.014, *p* = 0.007, *n* = 672). Thus, associations with hand length were not significant or were in the opposite direction than would be expected if 2D:4D were related to greater physical size.

Regarding height, regressions 13–18 had average 2D:4D, left 2D:4D, or right 2D:4D as the dependent variable and height as the independent variable that provided an index of physical size. Regressions 13, 15–17 had a significant main effect of sex (*d*s range from − 0.31 to − 0.65), but not of height (*d*s range from 0.01 to 0.02). Regressions 14 and 18 had a significant main effect of sex (*d* = − 0.47 and − 0.49, respectively) and a significant small positive main effect of height (*d* = 0.01 for both). Regressions 17 and 18 each had a significant interaction between the sex variable and height. In regression 17, the relationship between height and right 2D:4D was not significant for heterosexual men (*β* = − 0.001, *p* = 0.957, *n* = 275), whereas for heterosexual women the relationship was positive and significant (*β* = 0.031, *p* = 0.013, *n* = 275). In regression 18, the relationship between height and right 2D:4D was not significant for AMAB participants (*β* = 0.002, *p* = 0.733, *n* = 614), whereas for AFAB participants the relationship was positive and significant (*β* = 0.026, *p* = 0.001, *n* = 664). Thus, height was not significant or in the opposite direction than would be expected if lower 2D:4D were related to greater physical size.

Overall, we did not find support to suggest the sex difference in 2D:4D is explained by allometry in the current data. Instead, the findings are more in line with isometry. Thus, these results suggest it is reasonable to examine 2D:4D separately from other anthropometrics and to consider 2D:4D as a possible biomarker of processes influencing sex differentiation.

### Group differences in individual biomarkers

**Figure 1:**
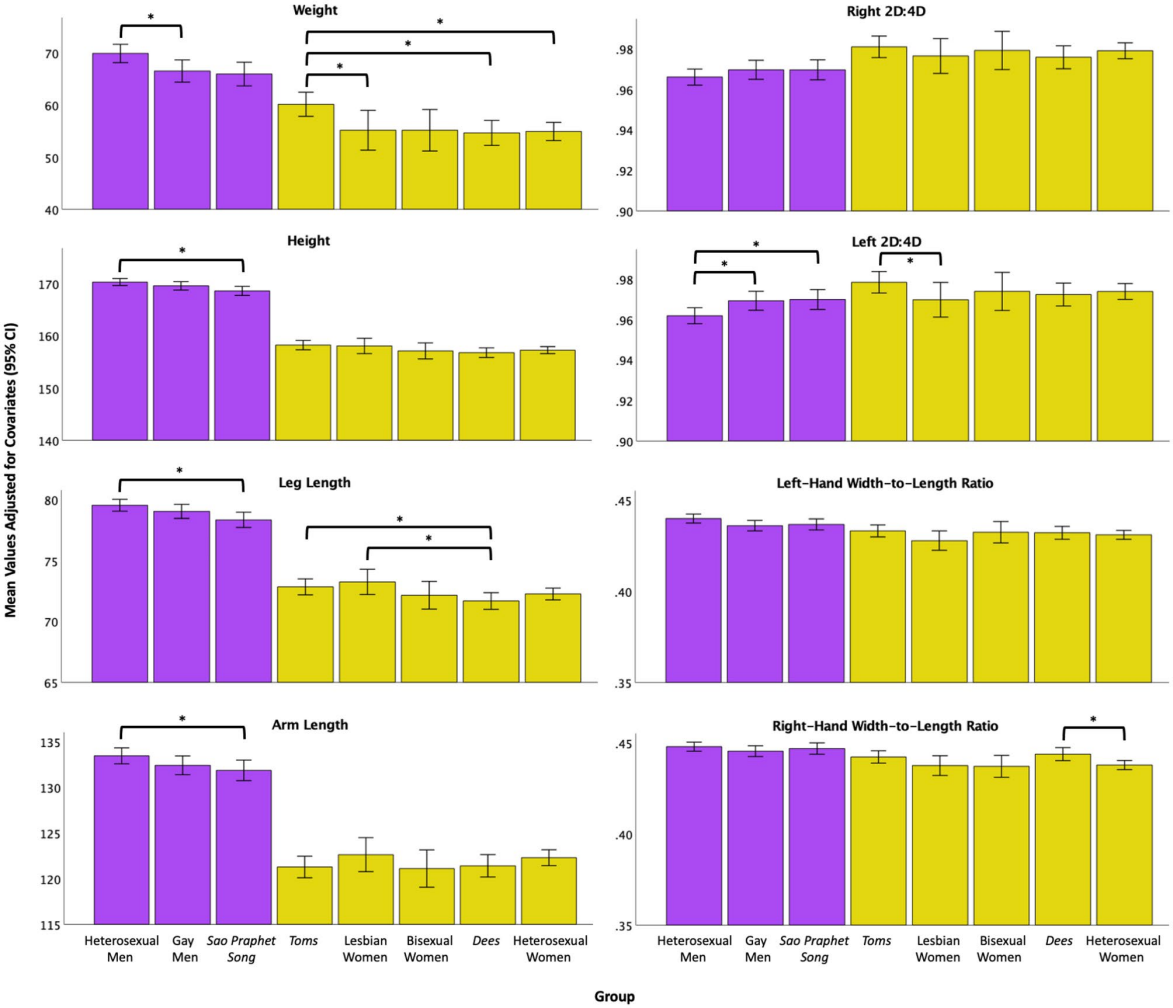
Group differences in mean biomarker values. Biomarkers are weight (kg) (*n* = 1316), height (cm) (*n* = 1317), leg length (cm) (*n* = 1260), arm length (cm) (*n* = 1255), left 2D:4D (*n* = 1307), right 2D:4D (*n* = 1306), left-hand width-to-length ratio (*n* = 1304), and right-hand width-to-length ratio (*n* = 1303). Values are adjusted for covariates (i.e., age and experimenter). Error bars represent 95% confidence intervals. All comparisons between heterosexual men and women are statistically significant at *p* < 0.001. Within-sex group differences are flagged by an asterisk (*) denoting *p* < 0.05.

Table 2 presents the descriptive statistics for the anthropometric variables by group. Figure 1 shows group comparisons for each biomarker. Full regression results are shown in the “Supporting Information” (Tables S14–S16) and only significant differences are reported here. Regressions were conducted in Mplus with age and experimenter added to control for these variables. Heterosexual men were significantly taller, heavier, had longer arms and legs, had higher left- and right-hand width-to-length ratios, and had lower left and right 2D:4D than heterosexual women (*p*s < 0.001, *d*s = |0.05–0.21|, see Table S14 for a list of Cohen’s *d* values and 95% CI for individual group comparisons)*.*

**Table 2:** Means and standard deviations for age and each anthropometric variable, by group.

	Het men	Gay men	*Sao praphet song*	*Toms*	Lesbian women	Bisexual women	*Dees*	Het women
**Age**								
*M*	25.95	23.53	25.27	26.56	24.12	24.40	24.52	28.26
*SD*	8.37	6.48	8.86	7.51	5.58	5.41	5.47	11.60
*n*	284	203	179	179	59	53	153	283
**Left 2D:4D**								
*M*	0.96	0.97	0.97	0.98	0.97	0.97	0.97	0.97
*SD*	0.03	0.03	0.03	0.03	0.03	0.03	0.04	0.03
*n*	278	190	172	163	56	46	133	280
**Right 2D:4D**								
*M*	0.96	0.97	0.97	0.98	0.98	0.98	0.98	0.98
*SD*	0.03	0.03	0.03	0.03	0.03	0.03	0.03	0.03
*n*	278	189	172	163	56	46	133	280
**Height**								
*M*	170.22	170.05	168.89	158.20	158.26	157.30	156.91	156.77
*SD*	5.97	6.11	5.73	4.73	4.53	5.22	6.09	5.86
*n*	279	188	159	169	56	51	145	279
**Leg length**								
*M*	79.46	79.28	78.48	72.86	73.34	72.25	71.80	72.03
*SD*	4.10	4.00	4.09	3.35	3.31	3.35	4.81	4.02
*n*	270	185	156	157	56	46	133	266
**Arm length**								
*M*	134.17	133.05	132.11	119.68	122.15	121.23	120.71	122.47
*SD*	6.84	7.26	8.16	6.54	6.77	6.99	7.90	7.65
*n*	269	185	152	157	56	46	133	266
**Left HWLR**								
*M*	0.44	0.43	0.44	0.44	0.43	0.43	0.43	0.43
*SD*	0.02	0.02	0.02	0.02	0.02	0.02	0.02	0.02
*n*	279	190	172	161	56	45	132	280
**Right HWLR**								
*M*	0.45	0.44	0.45	0.45	0.44	0.44	0.44	0.44
*SD*	0.02	0.02	0.02	0.02	0.02	0.02	0.02	0.02
*n*	278	190	172	161	56	45	132	280
**Weight**								
*M*	70.34	66.35	65.79	59.56	54.82	55.05	54.34	55.39
*SD*	15.20	16.02	17.98	14.58	8.72	13.76	14.50	11.69
*n*	280	188	159	169	56	51	145	277

Age is in years. Height, leg length, and arm length were measured in centimeters (cm) and weight was measured in kilograms (kg). Total *n*s range from 1264 (arm length) to 1393 (age). Values were calculated in SPSS.

*HWLR* hand width-to-length ratio, *Het* heterosexual.

Compared with gay men, heterosexual men weighed more (*p* = 0.041, *d* = − 0.20, 95% CI − 0.38 to − 0.01) and had lower left 2D:4D (*p* = 0.020, *d* = 0.24, 95% CI 0.05–0.42)*.* Compared with *sao praphet song*, heterosexual men were taller (*p* = 0.005, *d* = − 0.28, 95% CI − 0.48 to − 0.08), had longer arms (*p* = 0.027, *d* = − 0.23, 95% CI − 0.43 to − 0.03) and legs (*p* = 0.004, *d* = − 0.30, 95% CI − 0.50 to − 0.10), and had lower left 2D:4D (*p* = 0.010, *d* = 0.24, 95% CI = 0.05 to 0.44). There were no significant differences between gay men and *sao praphet song*.

Heterosexual women weighed less (*p* = 0.002, *d* = 0.34, 95% CI 0.14–0.53) than *toms* and had a lower right-hand width-to-length ratio than *dees* (*p* = 0.004, *d* = 0.30, 95% CI 0.09–0.51; i.e., hands that were less wide). *Toms* were also significantly heavier than lesbian women (*p* = 0.003, *d* = − 0.35, 95% CI − 0.66 to − 0.05) and *dees* (*p* = 0.002, *d* = − 0.40, 95% CI − 0.63 to − 0.17), had a larger left 2D:4D than lesbian women (*p* = 0.032, *d* = − 0.31, 95% CI − 0.62 to − 0.01), and had longer legs (*p* = 0.026, *d* = − 0.28, 95% CI − 0.52 to − 0.05) than *dees*. Lesbian women had longer legs than *dees* (*p* = 0.008, *d* = − 0.41, 95% CI − 0.72 to − 0.09).

## Discussion

This study examined a comprehensive set of putative anthropometric biomarkers of brain and behavioral sex differentiation in the largest and most diverse sample to date. Using EFA, the several biomarkers previously independently associated with sexual orientation and/or gender identity/expression were reduced to three factors: body size (i.e., height, leg length, arm length, and weight had the highest loadings), hand ratio (i.e., right and left hand width-to-length ratios had the highest loadings), and digit ratio (i.e., left and right 2D:4D had the highest loadings). However, we were not able to demonstrate that these factors were invariant across groups, indicating that the manner in which these biomarkers relate to one another varies in relation to sex, sexual orientation, and gender identity/expression. Further, contrary to the possibility that the sex difference in 2D:4D reflects allometry^38,39^, lower 2D:4D among individuals assigned male at birth than those assigned female at birth was not associated with greater physical size with respect to the average length of the second and fourth digits, hand length, or height. Based on these observations, one cannot conclude that the various biomarkers examined here reflect some latent biodevelopmental process(es) influencing sex differentiation. Instead, the present analysis suggests they may each provide unique insights. Thus, examining individual biomarkers should be considered as a tenable approach when investigating their associations with traits such as sex, sexual orientation, and gender identity/expression.

Importantly, heterosexual sex differences were found for each of the individual biomarkers. Consistent with prior research in the West^12,30,34,49^, compared with heterosexual women, heterosexual men were taller, heavier, had longer arms and legs, had wider hands, and lower 2D:4D than heterosexual women. Thus, we were able to confirm that these biomarkers were sex-differentiated as expected among Thais, suggesting they may be useful to study in relation to sexual orientation and gender identity/expression in this population. Within-sex differences, where found, were generally consistent with the notion that developmental processes underlying the biomarkers are associated with sexual orientation and/or gender identity/expression—although the patterns of group differences varied by biomarker and by birth-assigned sex.

Among individuals assigned male at birth, height, the long bones, weight, and left 2D:4D were associated with group differences. Specifically, heterosexual men were taller and had longer arms and legs than *sao praphet song*. These differences could reflect differential androgenic effects on long bone growth at the prenatal and/or pubertal window(s) of development. Long bone growth is influenced in part by androgens acting on androgen receptors, and epiphysial closure is influenced by estrogens^50,51^. Sex differences in these biomarkers generally appear during puberty, with surges in androgens influencing their development during prenatal and pubertal development^17^. Social factors (e.g., stress, nutrition, social roles) have also been related to the development of these biomarkers^17–19,36^. Processes such as these that are tied to height and the growth of long bones within the prenatal and pubertal windows may, therefore, be implicated in these group differences. Gay men were intermediate relative to heterosexual men and *sao praphet song* on these measures but did not differ significantly from either group. This pattern could reflect a “dosage” effect, but such an interpretation is tentative in the absence of significant group differences. In any case, it appears that differences from heterosexual men in height and long bone growth are more evident among the androphilic birth-assigned males who are more markedly feminine in their gender expression (i.e., *sao praphet song*) in the current sample. In this respect, our findings parallel those of prior Western research suggesting smaller body size among androphilic birth-assigned males who are more feminine^25,41^. Our findings, however, do not align with other Western research that has found that gay men were shorter than heterosexual men^20–22,24^, although degree of femininity was not assessed in these samples.

Regarding weight, gay men weighed less than heterosexual men, supporting some previous studies in the West^24,30^. Furthermore, *sao praphet song* weighed marginally less than heterosexual men (*p* = 0.051, see Table S16), which aligns with the shorter stature of *sao praphet song* relative to heterosexual men, and providing some support for one Western study of transgender same-sex attracted birth-assigned males^25^. Gay men did not show significant skeletal differences (i.e., height, long bone growth) but nevertheless weighed less than heterosexual men, suggesting that this group difference likely resulted from differences in muscle and fat mass. Indeed, compared with heterosexual men, gay men are more likely to use diet pills, diet, purge, fast to lose weight, be dissatisfied with their appearance, and experience eating disorders^52–54^. However, the extent to which such tendencies also apply to gay men in Thailand is not known, and so this interpretation should be considered speculative.

We also found that, compared with heterosexual men, left 2D:4D was significantly greater among both gay men and *sao praphet song*, who did not differ significantly from one another. Digit ratio is argued to develop mostly under the influence of prenatal androgen exposure, given fetal 2D:4D sex differences^55^, but also via some genetic influence^56^, with no evidence indicating sociocultural influences^10^. The pattern of group differences suggests these biodevelopmental processes are linked to a sexual orientation effect whereby androphilia in birth-assigned males is associated with more female-typical digit ratio, regardless of whether gender expression is relatively feminine. As such, this pattern runs contrary to a recent study that reported digit ratio was more female-typical among gay men who expressed feminine gender role behavior^57^. Further, meta-analyses have suggested digit ratio is more female-typical (vs. heterosexual men) among trans women^16^, but not gay men^12^ (but see findings with an adolescent sample^58^). Given the discrepant findings, it will be important for future research to continue to examine digit ratio in relation to both sexual orientation and gender identity/expression.

Of note, the present study found group differences for left, but not right, 2D:4D. Prior research similarly found sex assigned at birth and sexual orientation digit ratio differences are more apparent in one hand than the other (i.e., either non-existent or smaller in effect size in one hand); however, effects have typically been more apparent on the right, not left, hand^2,12,58,59^. An exception is a Japanese study of digit ratio that reported a male sexual orientation difference on the left, but not right, hand^60^. Thus, although we did not find associations between digit ratio and male sexual orientation in both hands, such effects are commonly found in only one hand, or are found to be stronger in one hand, and the group differences observed in left, but not right, 2D:4D among individuals assigned male at birth in the current study are consistent with research in another Asian population. Reasons why this might be the case for certain populations requires further research.

Regarding hand ratios, we did not find any group differences among heterosexual men, gay men, and *sao praphet song*. As such, our findings did not replicate those of an earlier study that reported lower hand width-to-length ratios among gay, compared with heterosexual, men^30^. Of the anthropometrics that have been studied in relation to male sexual orientation, hand ratios have been examined seldomly, and to our knowledge have not been examined in relation to gender identity/expression. Further research is needed to determine whether hand ratios are likely to be informative of biodevelopmental processes influencing male sexual orientation and/or gender identity/expression.

Among individuals assigned female at birth, group differences on the various biomarkers did not correspond to differences in androphilic vs. gynephilic sexual orientation but instead tended to correspond to gender-related differences. *Toms*, who are more masculine-presenting than the other birth-assigned female participants, were heavier than heterosexual women, lesbians, and *dees*, who are all more feminine-presenting. There is some research suggesting that more masculine (butch) lesbians have greater circulating testosterone levels, higher waist-to-hip ratios, more masculine digit ratios, and greater recalled childhood gender-nonconforming behavior than more feminine (femme) lesbians and heterosexual women^2,61,62^. Thus, the weight result may support some role of androgens in the development of *tom* identity, although there was no support for a dosage effect and interpretative caution is warranted given the only difference in 2D:4D is opposite to what would be expected (see below).

We also found that *toms* and lesbians had longer legs than *dees*. Despite these group differences in leg length, there were no differences among the birth-assigned female groups in height, corroborating most previous findings suggesting no relationship between height and sexual orientation in females^20–22^, cf.^23^ and suggesting leg length may be the more relevant proxy to consider among females (also see^30^). The leg length pattern observed here might reflect that more male-typical leg length has a biodevelopmental association with attraction to feminine partners (as displayed by *toms* and lesbians) vs. masculine partners (as displayed by *dees*). That said, if such were the case, one would expect heterosexual women to show shorter legs as well given they are, relatively speaking, attracted to masculine men. Alternatively, these leg length differences may be related to gender role expression. In Thailand, the gender role behavior of *toms* and lesbians appears to be relatively more masculine than that of heterosexual women and *dees*, and *dees* are less masculine than heterosexual women^48^. Thus, group differences in degree of masculine gender role expression might account for why only *dees* and not heterosexual women had shorter leg length than *toms* and lesbians.

There were also some unexpected group differences among individuals assigned female at birth. First, contrary to the prediction that more masculine groups would show lower 2D:4D, *toms* had higher left 2D:4D than lesbian women. That said, lack of support for our prediction is not necessarily out of step with other literature given recent meta-analytic findings suggesting no differences in 2D:4D between heterosexual women and trans men^16^. As such, processes contributing to digit ratio might not be related to the development of masculine identity among individuals assigned female at birth. Second, heterosexual women had lower (more feminine) right-hand width-to-length ratios than *dees*. Hand development is thought to be influenced by androgens modulating specific homeobox genes^63^, with some evidence also pointing to hand use during childhood^26,64^. Given *dees* were the only group of female gynephiles who had more masculine right-hand ratios than heterosexual women, this finding provided relatively weak evidence of female sexual orientation being influenced by such mechanisms.

Overall, the current pattern of results for individuals assigned female at birth may support some role of elevated androgens in the development of *toms* and lesbians—although there was no clear support for a dosage effect and interpretative caution is warranted given several null differences from female comparators (e.g., lack of difference with heterosexual women in leg length). In any case, the body of evidence for a biological basis to the development of sexual orientation and gender identity/expression in females cannot be discounted^2,65,66^ and the present findings suggest that gender-related factors should continue to be assessed in biomarker studies of sexual orientation and gender identity/expression in individuals assigned female at birth. Moreover, further research examining cross-cultural (in)consistencies in biomarker expression patterns among birth-assigned females is needed. Given previous suggestions that sexual orientation and gender identity/expression are more fluid and/or influenced by sociocultural factors among birth-assigned females than males^45,67–69^, one might expect more inconsistency in biomarker patterns across populations among the former. In other words, there are potentially more factors beyond biological mechanisms of sex differentiation contributing to female, compared with male, sexuality and gender identity/expression. If so, among female groups within particular populations, these alternative factors may to some extent obscure group differences related to biological mechanisms.

### Limitations

Biomarkers provide an indirect assessment of the mechanisms purported to influence sex differentiation of the brain and behavior, including sexual orientation and gender identity/expression. Future research examining how these biomarkers relate to brain sex differences or, where possible, longitudinal studies that measure these mechanisms directly and link them to later behavioral outcomes would be valuable. Previous studies have shown measurement of 2D:4D and sex differences in 2D:4D to be impacted by indirect (e.g., photocopies) versus direct measurement^70,71^. Given we employed a direct method of measurement, group differences may be impacted in future replications of this work. Also, an EFA approach to studying a comprehensive set of biomarkers in both sexes and in relation to both sexual orientation and gender diversity has not been reported in studies of Western samples, making it somewhat difficult to compare the current EFA-based results to previous studies conducted with Western samples. Thus, replication of this approach in a Western sample is an important future direction.

Convenience and non-random sampling, primarily in an urban center, was utilized in the current study, which limits generalizability of our findings to the general Thai population, rural Thailand, or other non-Western cultures. We note, however, that although representative samples would be worthwhile to collect, these tend to suffer from small sample sizes of sexually and gender diverse participants^21^. Also, although the final sample size was comparatively large for studies in this literature, group sizes were relatively smaller for bisexual women and lesbian women, which might reflect that it is more normative in Thai culture for same-sex attracted females to identify with the categories of *dees* or *toms* rather than the more Western-style categories of bisexual and lesbian^45^. Other groups could not be included due to their small sample size (i.e., bisexual men, transgender men). We were unable to examine biomarkers in *sao praphet song* primarily attracted to women, or *toms* primarily attracted to men. These gaps may be due to cultural norms surrounding the gender identification and sexual preferences of third/nonbinary gender individuals within Thai society^45^. More targeted approaches to recruiting may facilitate broader and larger samples in which such groups are represented and would benefit the aim of disentangling sexual orientation from gender identity.

## Conclusions

Our analysis of weight, height, leg length, arm length, digit ratio, and hand width-to-length ratio in a large and diverse Thai sample indicated that it is tenable to investigate these measures individually in relation to sex, sexual orientation, and gender identity/expression. Importantly, we were able to replicate expected heterosexual sex differences on these anthropometrics in a Thai population. Heterosexual men were taller, heavier, had longer arms and legs, had higher left- and right-hand width-to-length ratios and lower left and right 2D:4D than heterosexual women*.* Heterosexual men weighed more than gay men, and were taller, and had longer arms and legs than *sao praphet song*. Compared with both gay men and *sao praphet song*, heterosexual men had a lower, more male-typical, left 2D:4D*. Toms* were heavier than lesbian women, *dees*, and heterosexual women, and had longer legs than *dees*. Lesbian women also had longer legs than *dees*. The mechanisms underlying these anthropometrics are likely to influence psychological sex differences as well as the development of gender identity/expression and same-sex attraction. In particular, we found some support for the role of androgens in the development of same-sex attraction, and separately in the development of feminine gender identity/expression with the findings in birth-assigned males. For birth-assigned females, gender identity/expression seems to be more relevant than sexual orientation to group differences on anthropometrics, with a potential role of androgens. Future studies examining comprehensive sets of anthropometric measures in relation to sex, sexual orientation, and gender identity/expression simultaneously would allow for greater precision in our understanding of biodevelopmental influences on psychological sex differences as well as on sexual orientation and gender identity/expression.

## Methods

### Participants

Biomarkers were examined in 1404 heterosexual men, heterosexual women, gay men, lesbian women, bisexual women, *toms*, *dees*, and *sao praphet song* ages 18–72 years (*Mean* = 25.76, *SD* = 8.54, *n* = 1393; see Table 2 for *n* and age per group). Twelve bisexual men were excluded due to small group size. Also, seven transgender men were excluded. Although some previous research has characterized Thai *toms* and transgender men as belonging to the same gender identity category^44^, several transgender men in the present sample verbally communicated to the last author that they should not be considered equivalent to *toms*. As such, we opted to not include the transgender men alongside the *toms* and excluded them from the present analysis due to small sample size. Participants were recruited from May to July 2017 in Chiang Mai, Thailand, and the surrounding area via a network sampling procedure. The researchers approached people in public spaces (e.g., parks, shopping centers, village markets, along the street) to share information about the study and to invite them to participate. Those interested to participate made an appointment to do so and completed study measures in-person. Following participation, they were asked to share information about the study with others who might be interested to participate. This process continued throughout the period of participant recruitment. The rate of participation was > 90% among those invited by members of the study team—although it is unknown what percentage of those who were informed about the study from participants decided to participate as well. An honorarium of 300 Thai Baht was provided to each participant.

All methods were carried out in accordance with all relevant guidelines and regulations. All procedures were approved by the Research Ethics Board at the University of Toronto (#35931) and performed in accordance with this approval. Informed consent was obtained from all participants by DPV and a Thai research assistant. DPV and LAC received permissions from the Royal Thai Embassy to conduct this research in Thailand.

### Measures

All study measures were translated and back-translated by two individuals fluent in English and Thai. Physical measurements were performed by LAC or DPV. Questionnaire and interview measures were collected by Thai research assistants, fluent in both Thai and English, using a standard questionnaire and interview. Specifically, participants completed a questionnaire that included questions about birth-assigned sex, gender/sexual orientation identity, and sexual attractions; their responses to these questions were then subsequently reviewed with them orally during an interview with a Thai research assistant to confirm or, if necessary, clarify responses. All questionnaires and interviews took place under the supervision of DPV.

### Birth-assigned sex

Participants reported sex at birth, with options of “male,” “female,” and a free response option of “ambiguous/other,” which did not apply for any participants.

### Gender/sexual orientation identity

Participants were grouped on the basis of birth-assigned sex and through questionnaires and interviews about their identities and sexual attractions. Those assigned male at birth with a male or masculine identity/presentation and predominant or exclusive attraction to women were classified as heterosexual men, whereas those with predominant or exclusive attraction to men were classified as gay men. Those assigned male at birth who identified as a woman or *sao praphet song*, with a feminine presentation and predominant or exclusive attraction to men were classified as *sao praphet song*. Those assigned female at birth who identified as women and/or with a feminine presentation and predominant or exclusive attraction to men were classified as heterosexual women, whereas lesbian women were those with predominant or exclusive attraction to women, bisexual women were those with attraction to both sexes, and *dees* were predominantly or exclusively attracted to *toms*. Birth-assigned females were classified as *toms* if they identified as *toms*, presented/identified as masculine, and had predominant or exclusive attraction to women or *dees.* The “Supporting Information” provides further information regarding sexual attractions.

### Anthropometric measures

Objects that interfered with anthropometric measurements (e.g., resting hands flat on a surface), including shoes, heavy clothes, and objects from pockets were removed. Measurements were recorded to the nearest decimal, or two decimals for those measured using digital calipers.

*Digit ratio* (2D:4D) was calculated as the ratio of the length of the index finger (2D) to the ring finger (4D) for each hand. Hands were positioned flat on a table, with palms up and fingers together. Fingers were measured in millimeters (mm) using digital calipers. The most proximal crease for each finger was used as a starting point.

*Height* was measured in centimeters (cm). Standing against a wall, a carpenter’s square was placed on the top of the head while in the Frankfurt position. The wall was marked at that position, and the space between the floor and the marking was measured using a tape measure.

*Leg length* was calculated by subtracting seated height (minus the height of an unadjusted stool) from standing height in cm. Seated height was measured with feet touching the floor and lower back touching the wall (similar to^21^). A right triangle rested on the vertex of the head, in the Frankfurt position, and the wall. The wall was marked and the space between the floor and the marking was measured using a tape measure.

*Arm length* was calculated by subtracting biacromial breadth from arm span in cm and reflects both right and left arms. Biacromial breadth (i.e., distance between the most lateral extent of the two scapulae) was determined by palpation of the left and right acromial processes and then measured with a tape measure. Arm span was measured by asking participants to stretch out their arms, adjusting them to a level height using a carpenter’s level, marking the extent of the fingertips against the wall, and then measuring the distance between the two points on the wall with a tape measure.

*Hand width-to-length ratios* were calculated by dividing the hand width by the hand length for each hand. Hand widths and lengths were measured on the ventral (palm) surface^30^. A tape measure was extended from the most distal crease of the hand to the tip of the third digit to measure hand length in cm. Hand length was converted into mm before ratio calculation. Digital calipers were extended from the most lateral point of the second metacarpal to the most lateral surface of the fifth metacarpal to measure hand width in mm.

*Weight* was measured using a digital scale in kilograms.

### Statistical analyses

Although 1404 participants were included in analyses, not all participants had complete data for all measures (e.g., certain biomarkers could not be measured due to physical deformities, equipment malfunctions). For the biomarkers specifically, missing data ranged from 5.6% (*n* = 78) to 10.0% (*n* = 140). Thus, not all participants were included in the results of all analyses. In regressions, complete case analysis was used to deal with missing data and we indicate sample sizes for each analysis. Analyses were run in Mplus (version 8.6^72^) using maximum likelihood estimation with robust standard errors (MLR) to account for some non-normality displayed with the age and weight variables. For correlation analyses that included a dichotomous variable, the diagonal weighted least squares (WLSMV) estimator was used. Some analyses and calculation of some descriptive statistics were conducted in SPSS (version 27) and we note where this occurred. Data files and code used in analyses can be found on Scholar’s Portal Dataverse^73^.

Exploratory factor analysis (EFA) with 1–3 factors using oblique geomin rotation was performed on all biomarkers (i.e., left and right 2D:4D, height, leg length, arm length, left- and right-hand width-to-length ratio, weight). Factor loadings of 0.40 or greater were interpreted. To deal with missing data, MLR was used whereby participants with at least one biomarker were included in the EFA.

To examine allometry in 2D:4D following the method described by Forstmeier^39^, regressions were performed comparing heterosexual men (coded 1) with heterosexual women (coded 0) (*n* = 571), and comparing all individuals assigned male at birth (AMAB; coded 1) (i.e., heterosexual men, gay men, *sao praphet song*) with all individuals assigned female at birth (AFAB; coded 0) (i.e., heterosexual women, *toms*, lesbian women, bisexual women, *dees*) (*N* = 1404), using dichotomized variables as the independent variables. The various physical size variables (see Table 1) were also independent variables. Average 2D:4D, left 2D:4D, or right 2D:4D were the dependent variables. Covariates were age and experimenter; the “Supporting Information” summarizes results related to the covariates. The first set of participants (i.e., heterosexual participants only) limits the analyses to groups assumed to be less affected by variation in mechanisms underlying their development and the second set allows for examination of a larger sample.

A first set of regressions provided tests of main effects, and a second set of regressions tested the interaction between the sex variable and the various physical size variables. Two sets of regressions were utilized to estimate the main effects independent of their possible interaction, akin to a regression analysis with variables entered in blocks. In the first set of regressions, the sex variable was entered along with average finger length (i.e., average of right 2D, right 4D, left 2D, and left 4D; as in^39^), average left hand finger length (i.e., average of left 2D and left 4D), average right hand finger length (i.e., average of right 2D and right 4D), average hand length (i.e., average of right hand length and left hand length), right hand length, left hand length, or height. The dependent variable was the average 2D:4D (i.e., average of right 2D:4D and left 2D:4D), left 2D:4D, or right 2D:4D. Then, in a second set of regressions, the interaction between the sex variable and the finger length, hand length, or height variable was added to the other variables listed above. If isometry is present, there should be a significant main effect of sex and a non-significant effect for the measure of physical size. If allometry is present, there should be a small and significant effect of sex and a large and significant negative effect for the measure of physical size. An interaction between sex and measure of physical size would indicate allometry might apply more so to one sex.

To examine group differences in biomarkers, regressions were performed within the heterosexual participants (i.e., heterosexual men, heterosexual women), within AMAB groups (i.e., heterosexual men, gay men, *sao praphet song*), and within AFAB groups (i.e., heterosexual women, *toms*, lesbian women, bisexual women, *dees*), using dummy coded variables as the independent variables. Individual biomarkers were dependent variables. Covariates were age and experimenter. The “Supporting Information” summarizes results related to the covariates. Also, see the “Supporting Information” for EFA details, calculation of Cohen’s *d* for effect sizes, full details on analyses of group comparisons on each biomarker, and additional analyses (e.g., evaluation of other variables, such as hormone use and education background, as potential covariates).

## Supplementary Information

Supplementary Information.
