# Supplementary material for: An anthropometric study of sexual orientation and gender identity in Thailand
Source: Sci Rep. 2021 Sep 16;11:18432. doi: 10.1038/s41598-021-97845-9 (PMC8445993; doi:10.1038/s41598-021-97845-9)
Supplement: Supplementary file 1 — Supplementary Information. [file 41598_2021_97845_MOESM1_ESM.pdf]

Scientific Reports

Supporting Information for:

An anthropometric study of sexual orientation and gender identity in Thailand

Malvina N. Skorska, Lindsay A. Coome, Diana E. Peragine, Madison Aitken, and Doug P. VanderLaan

Address correspondence to D. P. VanderLaan
E-mail: doug.vanderlaan@utoronto.ca

This file includes:

Supplementary text
Tables S1 to S16
SI References

Exploratory Factor Analysis Across Groups

An exploratory factor analysis (EFA) was conducted on the eight biomarkers using oblique rotation (geomin) for 1356 participants using Mplus. Descriptive statistics are in Table S1 and correlations among the biomarkers, which ranged from $r = |.912|$ to $r = |.020|$, are in Table S2. Given eight biomarkers, one, two, and three factor solutions were tested. The one-factor solution did not have good fit (root mean square error of approximation (RMSEA) = .25, comparative fit index (CFI) = .49, $\chi^2(20) = 1714.14$, $p < .001$, standardized root mean squared residual (SRMR) = .17) and the two-factor solution had better fit (RMSEA = .15, CFI = .87, $\chi^2(13) = 392.39$, $p < .001$, SRMR = .08). The three-factor solution had the best fit (RMSEA = .08, CFI = .98, $\chi^2(7) = 70.57$, $p < .001$, SRMR = .01). Factor loadings are available in Table S3 for the three-factor solution. Factor 1 (Body Size) was significantly correlated with Factor 2 (Hand Ratio) ($r = -.228$, $p < .05$; exact p-values are not provided) and 3 (Digit Ratio) ($r = -.094$, $p < .05$), which were not significantly correlated with each other ($r = .131$, $p > .05$). Thus, the three-factor solution provided a good fit to the data across all groups.

Table S1. Descriptive statistics for biomarkers across group.

	n	Mean	Variance	Skewness	Kurtosis	Min	Max
Weight (kg)	1325	61.731	251.466	1.505	3.867	31.8	172.0
Height (cm)	1326	163.217	72.042	0.095	-0.577	137.1	187.7
Leg length (cm)	1269	75.606	27.854	-0.047	0.251	53.2	91.3
Arm length (cm)	1264	127.077	90.183	-0.409	1.337	76.0	159.9
Left 2D:4D	1318	97.048	10.853	0.306	1.359	84.0	118.0
Right 2D:4D	1317	97.349	11.196	0.383	0.795	86.0	114.0
Left HWLR	1315	43.477	4.447	0.232	0.646	37.0	54.0
Right HWLR	1314	44.338	4.728	0.218	0.291	38.0	53.0

Note. HWLR = hand width-to-length ratio; Min = minimum; Max = maximum.

Table S2. Correlations among the biomarkers.

		Weight	Height	Leg length	Arm length	Left 2D:4D	Right 2D:4D	Left HWLR
Height	*r*	**.467**						
	p	**< .001**						
Leg length	*r*	**.332**	**.912**					
	p	**< .001**	**< .001**					
Arm length	*r*	**.295**	**.818**	**.801**				
	p	**< .001**	**< .001**	**< .001**				
Left 2D:4D	*r*	.028	**-.061**	**-.057**	-.039			
	p	.315	**.028**	**.037**	.137			
Right 2D:4D	*r*	.020	**-.076**	**-.070**	**-.097**	**.504**		
	p	.503	**.006**	**.013**	**< .001**	**< .001**		
Left HWLR	*r*	**.327**	**-.106**	**-.177**	**-.227**	-.037	-.020	
	p	**< .001**	**< .001**	**< .001**	**< .001**	.162	.491	
Right HWLR	*r*	**.311**	**-.119**	**-.191**	**-.243**	**-.055**	-.048	**.792**
	p	**< .001**	**< .001**	**< .001**	**< .001**	**.047**	.097	**< .001**

Note. HWLR = hand width-to-length ratio. $n = 1356$. **Bold**, $p < .05$. Correlations were conducted in Mplus.

Table S3. Geomin rotated loadings from exploratory factor analysis.

	Factor 1: Body Size	Factor 2: Hand Ratio	Factor 3: Digit Ratio
Weight	**0.537***	**0.490***	0.019*
Height	**1.004***	0.089*	-0.008
Leg length	**0.924***	-0.018*	-0.001
Arm length	**0.819***	-0.097*	-0.012
Left 2D:4D	-0.003	-0.012	**0.638***
Right 2D:4D	-0.003	0.012	**0.788***
Left HWLR	0.008	**0.896***	-0.157
Right HWLR	-0.010	**0.893***	-0.192

Note. HWLR = hand width-to-length ratio. Loadings > .40 are in **bold**. $n = 1356$. *$p < .05$.

Exploratory Factor Analysis by Group

To investigate measurement invariance, the first step is to conduct the EFA within each group. The factor structure and model fit should replicate to proceed to the next step (i.e., testing various versions of the model while increasing the number of constraints). Table S4 shows the factor loadings for the three-factor solution for EFAs conducted within each group using Mplus. Table S5 shows the model fit statistics accompanying each of these EFAs. Although model fit was acceptable in heterosexual men, gay men, and heterosexual women, fit statistics for *sao praphet song* and *toms* suggested unacceptable model fit, models did not converge for lesbian women, bisexual women, or *dees*, and geomin rotated loadings were not provided for heterosexual women. In addition, local minimum solutions were identified for heterosexual men, gay men, *sao praphet song*, and *toms*. Based on these initial subgroup results, we were unable to replicate the factor structure consistently across groups or demonstrate measurement invariance across groups.

Table S4: Geomin rotated loadings for the three-factor solution, by group.

	Weight	Height	Leg length	Arm length	Left 2D:4D	Right 2D:4D	Left HWLR	Right HWLR
				Heterosexual men[$], *n* = 280				
F1	0.466*	0.980*	0.897*	0.676*	-0.015	0.014	-0.015	-0.068
F2	0.429*	0.010	-0.064	-0.203*	-0.049	0.022	0.887*	0.843*
F3	0.161*	0.054	-0.026	-0.018	0.750*	0.626*	-0.010	-0.057
				Gay men[$], *n* = 193				
F1	0.484*	0.993*	0.868*	0.725*	-0.007	0.070	0.001	-0.005
F2	0.017*	-0.074	-0.027	0.017	1.165*	0.454*	-0.214	-0.225
F3	0.565*	0.012	-0.104	-0.183*	-0.003	0.033	0.898*	0.890*
				Sao praphet song[$], *n* = 174				
F1	0.416*	0.994*	0.823*	0.524*	0.008	-0.096	0.008	-0.062
F2	0.450*	0.007	-0.155*	-0.378*	0.088	-0.015	0.914*	0.830*
F3	0.043	0.049	-0.031	-0.006	0.773*	0.629*	-0.109	-0.054
				Toms[$], *n* = 175				
F1	0.366*	1.016*	0.857*	0.706*	-0.070	0.009	-0.021	0.002
F2	0.640*	0.202	-0.007	-0.053	-0.007	0.152	0.874*	0.940*
F3	0.022	-0.009	-0.015	0.062	0.758*	0.735*	-0.087	-0.231
				Lesbian women, *n* = 56				
Model did not converge								
				Bisexual women, *n* = 51				
Model did not converge								
				Dees, *n* = 145				
Model did not converge								
				Heterosexual women[^], *n* = 282				
Optimal rotation is not sufficiently identified								

Note. F1 = Factor 1, F2 = Factor 2, F3 = Factor 3, HWLR = hand width-to-length ratio. [$]Two or more geomin rotated loadings were provided with two or more local minimum values. [^]A warning regarding this exploratory factor analysis indicates that standard errors could not be computed for the three-factor solution, and the optimal rotation is not sufficiently identified. A factor structure is provided (not shown), but geomin rotated loadings were not provided. *$p < .05$ (for the first set of loadings if multiple loadings were provided). Exploratory factor analyses were conducted in Mplus.

Table S5: Model fit statistics for the three-factor solution, by group.

	RMSEA	CFI	SRMR	$\chi^2(7)$, p-value
Heterosexual men	**.07**	.98	.01	16.96, .018
Gay men	**.06**	.99	.01	11.36, .124
Sao praphet song	**.16**	.91	.02	39.11, < .001
Toms	**.18**	.92	.03	46.46, < .001
Lesbian women		Model did not converge		
Bisexual women		Model did not converge		
Dees		Model did not converge		
Heterosexual women	.07	**.99**	.03	15.83, .027

Note. RMSEA = root mean square error of approximation, CFI = comparative fit index, SRMR = standardized root mean squared residual. Exploratory factor analyses were conducted in Mplus.

Cohen's *d* Calculations

Cohen's *d* (and 95% CIs) were calculated using unstandardized B's from regressions (that had covariates in the model, and thus reflect the effect adjusted for covariates), standard deviation of the dependent variable, and sample sizes for relevant groups using the online calculator found at https://www.campbellcollaboration.org/escalc/html/EffectSizeCalculator-SMD21.php. In the Main Text and Supporting Information, we only report Cohen's *d* for the main results. We include a file, "CohensDThaiBiomarker_rev2.xlsx," on Scholar's Portal Dataverse [1] that includes all necessary information to calculate Cohen's *d* (and 95% CI) for other comparisons included in the Supporting Information for those who are interested.

Sexual Attractions of Participants

Participants were asked to report their sexual attractions to four groups of individuals over the previous 12 months: men (i.e., all birth-assigned male categories but *sao praphet song*), women (i.e., all birth-assigned female categories but *toms*), *sao praphet song*, and *toms*. A Kinsey-style 7-point scale was used to assess sexual attraction, where 0 represented, "*None of my sexual attractions*" and 6 represented, "*All of my sexual attractions.*" Responses to the four groups needed to sum to 6 because "6" represented all sexual attractions over the past year. Mean ratings are shown in Table S6 and correspond to our criteria for grouping participants (e.g., gay men and heterosexual women have high ratings for attraction toward men, but low ratings for attraction towards the other groups; heterosexual men and lesbian women have high ratings for attraction toward women, but low ratings for attraction towards the other groups).

Table S6. Mean (standard deviation) of sexual attractions to men, women, *sao praphet song*, and *toms*, by group.

Group	*n*	Attraction toward men	Attraction toward women	Attraction toward *sao praphet song*	Attraction toward *toms*
Heterosexual men	286	0.07 (0.53)	5.74 (0.74)	0.12 (0.37)	0.08 (0.33)
Gay men[a]	204	5.50 (1.11)	0.26 (0.69)	0.20 (0.81)	0.03 (0.20)
Sao praphet song	181	5.83 (0.72)	0.05 (0.35)	0.03 (0.23)	0.08 (0.46)
Toms	181	0.09 (0.43)	5.71 (0.88)	0.11 (0.39)	0.09 (0.52)
Lesbian women	59	0.42 (0.65)	4.51 (1.60)	0.14 (0.43)	0.93 (1.47)
Bisexual women	53	2.70 (1.27)	1.74 (1.44)	0.19 (0.56)	1.38 (1.69)
Dees	154	0.40 (0.79)	0.47 (1.06)	0.02 (0.14)	5.11 (1.36)
Heterosexual women[b]	282	5.57 (0.90)	0.24 (0.64)	0.03 (0.17)	0.15 (0.56)

Note. [a]Data missing for *n* = 1 participant. [b]Data missing for *n* = 3 participants. *N* = 1404. This table was constructed using SPSS.

Covariates

Age and experimenter

Regressions in Mplus using dummy coded variables were conducted with each group as the reference category. Heterosexual women were significantly older than all other groups except *toms*, and heterosexual men were significantly younger than heterosexual women, but older than gay men and *sao praphet song* (see Table S7 for all pairwise comparisons). Age was significantly related to height, leg length, arm length, right 2D:4D, and left- and right-hand width-to-length ratio (Table S8). Experimenter was associated with group, $\chi^2(7) = 203.511$, $p < .001$ ($N = 1404$; this analysis was conducted in SPSS), given DPV measured more of the male participants and more of the heterosexual women, whereas LAC measured more of the other female groups. As a result, experimenter, with DPV coded as 1 and LAC coded as 2, was related to weight, height, leg length, arm length, right 2D:4D, and left- and right-hand width-to-length ratio (Table S8). Age was related to experimenter, $r = .092$, $p = .006$, $N = 1404$. Thus, age and experimenter were statistically controlled in most analyses (unless otherwise specified).

Table S7. Results of regressions with age as the dependent variable and dummy coded group as the independent variables.

Group 1 (coded 0)	Group 2 (coded 1)	*β*	*SE*	*p*
Heterosexual men	Gay men	-0.284	.078	**< .001**
	Sao praphet song	-0.080	.097	.410
	Toms	0.071	.088	.416
	Lesbian Women	-0.215	.101	**.034**
	Bisexual Women	-0.182	.103	.076
	Dees	-0.168	.077	**.028**
	Heterosexual women	0.270	.097	**.005**
Gay men	*Sao praphet song*	0.250	.093	**.028**
	Toms	0.356	.085	**< .001**
	Lesbian Women	0.069	.100	.488
	Bisexual Women	0.102	.102	.317
	Dees	0.116	.075	.121
	Heterosexual women	0.554	.091	**< .001**
Sao praphet song	*Toms*	0.151	.102	.138
	Lesbian Women	-0.135	.114	.234
	Bisexual Women	-0.103	.115	.372
	Dees	-0.089	.092	.337
	Heterosexual women	0.349	.109	**.001**
Toms	Lesbian Women	-0.286	.106	**.007**
	Bisexual Women	-0.254	.108	**.018**
	Dees	-0.240	.083	**.004**
	Heterosexual women	0.198	.102	.051
Lesbian women	Bisexual Women	0.032	.121	.788
	Dees	0.047	.099	.638
	Heterosexual women	0.485	.111	**< .001**
Bisexual women	*Dees*	0.014	.100	.889
	Heterosexual women	0.452	.113	**< .001**
Dees	Heterosexual women	0.438	.089	**< .001**

Note. Only non-redundant pairwise comparisons are included. **Bold**, $p < .05$. $n = 1393$. Analyses conducted in Mplus.

Table S8. Correlations between age, experimenter, and biomarkers.

	Weight	Height	Leg length	Arm length	Left 2D:4D	Right 2D:4D	Left HWLR	Right HWLR
Age	.043	-.247	-.225	-.215	.006	-.057	.147	.176
	.113	**< .001**	**< .001**	**< .001**	.807	**.028**	**< .001**	**< .001**
Exp.	-.130	-.190	-.182	-.425	-.006	.216	.232	.225
	**< .001**	**< .001**	**< .001**	**< .001**	.853	**< .001**	**< .001**	**< .001**

Note. Exp. = Experimenter; HWLR = hand width-to-length ratio. $N = 1404$. For experimenter, DPV was coded 1 and LAC was coded 2. Correlations conducted in Mplus.

Hormone use in sao praphet song and gay men

Sao praphet song are likely to use exogenous feminizing hormones—typically oral contraceptives—with an onset of use in mid-adolescence, on average [2,3]. Comparatively, rates of hormone use among *toms* are low [2]. The widespread use of exogenous feminizing hormones among *sao praphet song* and possibly gay men has implications for the interpretation of our measured biomarkers. It is unlikely that 2D:4D would be affected by exogenous hormone use in adolescence given digit ratio is established early in prenatal development under the influence of sex hormones [4]. However, with respect to height, sex hormones play a pivotal role in skeletal development during adolescence. Thus, it is important to consider exogenous hormone use among individuals in our sample especially when comparing measures of body or hand size.

Use of exogenous hormones was measured for all participants via self-report. Participants were asked whether they had ever used exogenous hormones and for those who indicated they had ever used exogenous hormones, they were asked to provide the age (in years) at first use. Participants were then asked whether they were using hormones anytime within the past six months. Hormone usage was only examined for gay men and *sao praphet song*, given low usage rates in the other groups. Specifically, one heterosexual man indicated he ever used hormones and because of the low cell size and because the majority of heterosexual men did not ever use exogenous hormones, heterosexual men were not included in analyses. Within female groups, some had low cell sizes (i.e., three *toms* ever used exogenous hormones and two lesbian women ever used exogenous hormones) and thus exogenous use of hormones among females was not investigated in relation to biomarkers.

Thirty-seven gay men (18.0%) and 157 *sao praphet song* (86.7%) reported ever using exogenous hormones, with *sao praphet song* more likely to use exogenous hormones than gay men, $\chi^2(1) = 181.44$, $p < .001$ ($n = 386$; this analysis was conducted in SPSS). Correlation analyses revealed that weight was significantly related to using hormones within the past six months. All other correlations between exogenous hormone use or age at first exogenous hormone use and the biomarkers were not significant (see Table S9). Given the significant association between hormone use in the past six months and weight, we ran a regression examining differences in weight between gay men and *sao praphet song* controlling for age, experimenter, and hormone use. The difference in weight between gay men and *sao praphet song* was still not significant, $\beta = -0.290$, $SE = .203$, $p = .152$, $n = 170$, similar to the lack of group difference in the main analyses (i.e., not controlling for hormone use). Current hormone use was negatively associated with weight ($p = .018$), but age and experimenter were not (ps > .076). Thus, exogenous hormone use or age at first exogenous hormone use did not impact results and we did not control for exogenous hormone use or age at first exogenous hormone use in the main analyses.

Table S9. Correlations between hormone use variables and biomarkers in gay men and *sao praphet song*.

		Weight	Height	Leg Length	Arm Length	Left 2D:4D	Right 2D:4D	Left HWLR	Right HWLR
Age of First Hormone Use[a]	*r*	.020	.011	-.065	-.256	.082	-.032	.053	.118
	p	.796	.900	.459	.241	.171	.573	.448	.185
Ever Used Hormones[b,c]	*r*	-.078	-.044	-.025	.061	-.049	.060	-.071	-.066
	p	.164	.509	.715	.373	.438	.362	.280	.314
Used Hormones Within the Past Six Months[a,c]	*r*	**-.188**	-.039	.036	.082	-.027	-.017	-.075	-.015
	p	**.036**	.685	.699	.437	.794	.868	.399	.869

Note. HWLR = hand width-to-length ratio. [a] *n* = 374; [b]*n* = 386; [c]Dichotomous variables with "no" coded 1 and "yes" coded 2. Correlations conducted in Mplus.

Education and income

Participants answered questions about the highest level of education completed and their income (see Table S10 for sample size for each category and for the coding scheme for dichotomizing each variable). Education and socioeconomic status have been included as covariates in some previous studies of sexual orientation and height [5]; however, other studies investigating the biomarkers examined in the current study have not used education and socioeconomic status as covariates, such as studies investigating 2D:4D. Thus, we decided not to include them as the main covariates of interest but to examine if they impact the main group differences only if it is shown that they are related to the biomarkers and to group.

The biomarkers were significantly related to education (for height, leg length, arm length, right 2D:4D, and right-hand width-to-length ratio) and were significantly related to income level (for height, leg length, and arm length) (Table S11). Furthermore, group was associated with education ($\chi^2(7) = 40.76$, $p < .001$, $n = 1391$; this analysis was conducted in SPSS) and income ($\chi^2(7) = 25.63$, $p = .001$, $n = 1385$; this analysis was conducted in SPSS).

After running the main analyses (i.e., only for those biomarkers that were correlated with education or income, and for those group comparisons that were significant; see Table S12) with each of these variables (first dichotomized according to Table S10) as additional covariates (i.e., age and experimenter were also included as covariates), the following results were affected: right-hand width-to-length ratio was no longer significantly different between heterosexual women and *toms* when controlling for education; right-hand width-to-length ratio was no longer significantly different between lesbian women and *dees* when controlling for education; height was no longer significantly different between *toms* and *dees* when controlling for education. Thus, we did not interpret these findings in the main analyses but present them in the SI for completeness. All other differences investigated remained statistically significant (Table S12).

For the regressions summarized in Table S12, education was not significant for all comparisons (ps > .056). Income was significantly positively associated with height in the comparison between *toms* and *dees* ($p = .014$) and was significantly negatively associated with arm length in the comparison between heterosexual men and *sao praphet song* ($p = .039$). For all other comparisons income was not significant (ps > .093).

In regressions controlling for education, age was significantly positively associated with right-hand width-to-length ratio in all instances (ps < .001) and was significantly negatively associated with every other biomarker (ps ≤ .003). Experimenter was significantly positively associated with right-hand width-to-length ratio in all instances (ps < .001) and was significantly negatively associated with arm length and with right 2D:4D in the comparison between heterosexual men and heterosexual women, and with arm length in the comparison between heterosexual men and *sao praphet song* (ps ≤ .003). In all other instances, experimenter was not significant (ps > .268). These same patterns emerged in regressions controlling for income.

Table S10. Sample size and coding for education and income.

Variable	*n*	Category	Dichotomized Variable Coding	*n*
Education	48	Less than primary or primary	0	
	91	Some high school	0	
	231	High school diploma	0	
	488	Some college/trade school/university	0	858
	110	College diploma or trade school complete	1	
	397	University degree complete (Bachelor's)	1	
	26	University degree complete (Master's/PhD/Professional degree)	1	533
Income	511	Less than 5000 Thai Baht	0	
	390	5000-9999 Thai Baht	0	901
	326	10000-14999 Thai Baht	1	
	92	15000-19999 Thai Baht	1	
	40	20000-29999 Thai Baht	1	
	26	30000 Thai Baht or more	1	484

Note. N = 1404. For education, data were missing for 13 participants. For income, data were missing for 19 participants. SPSS was used to create this table.

Table S11. Correlations between education, income, and the biomarkers.

		Weight	Height	Leg Length	Arm Length	Left 2D:4D	Right 2D:4D	Left HWLR	Right HWLR
Income	r	-.035	**-.141**	**-.135**	**-.128**	-.010	-.004	.008	.064
	p	.342	**< .001**	**< .001**	**< .001**	.789	.905	.828	.073
Education	r	-.027	**-.150**	**-.139**	**-.174**	.001	**.070**	.036	**.072**
	p	.445	**< .001**	**< .001**	**< .001**	.972	**.044**	.319	**.044**

Note. HWLR = hand width-to-length ratio. N = 1404. See Table S10 for dichotomous coding of education and income. Correlations were conducted in Mplus.

Table S12. Main results of regressions for significant comparisons in the main analyses controlling for education and controlling for income.

Controlling for education		*n*	*β*	*p*
Height	Heterosexual men vs heterosexual women	549	-.209	**< .001**
Arm length	Heterosexual men vs heterosexual women	526	-.171	**< .001**
Leg length	Heterosexual men vs heterosexual women	527	-.190	**< .001**
Right 2D:4D	Heterosexual men vs heterosexual women	549	.054	**< .001**
Right hand HWLR	Heterosexual men vs heterosexual women	549	-.068	**< .001**
Right hand HWLR	Heterosexual women vs *toms*	666	.212	.051
Right hand HWLR	Heterosexual women vs *dees*	666	.287	**.006**
Right hand HWLR	Lesbian women vs *dees*	666	.298	.058
Leg length	Lesbian women vs *dees*	650	-.376	**.012**
Leg length	*Toms* vs *dees*	650	-.255	**.044**
Height	*Toms* vs *dees*	692	-.222	.053
Height	Heterosexual men vs *sao praphet song*	615	-.276	**.005**
Arm length	Heterosexual men vs *sao praphet song*	595	-.220	**.033**
Leg length	Heterosexual men vs *sao praphet song*	600	-.295	**.004**
Controlling for income				
Height	Heterosexual men vs heterosexual women	545	-.208	**< .001**
Arm length	Heterosexual men vs heterosexual women	524	-.170	**< .001**
Leg length	Heterosexual men vs heterosexual women	525	-.189	**< .001**
Leg length	Lesbian women vs *dees*	643	-.372	**.015**
Leg length	*Toms* vs *dees*	643	-.254	**.047**
Height	*Toms* vs *dees*	685	-.231	**.046**
Height	Heterosexual men vs *sao praphet song*	615	-.275	**.005**
Arm length	Heterosexual men vs *sao praphet song*	597	-.220	**.031**
Leg length	Heterosexual men vs *sao praphet song*	602	-.295	**.004**

Note. HWLR = hand width-to-length ratio. The first group listed is coded 0 and the second group listed is coded 1. Age and experimenter were also controlled (results not shown). Regressions were conducted in Mplus.

Additional Details from Allometry Analyses

Table S13 provides the mean and standard deviation for each 2D:4D and physical size variable included in the allometry analyses, by the four groups included in those analyses.

Age and experimenter were controlled in the allometry analyses. Experimenter was significantly positively associated with the outcome variable in the following regressions (see Table 1 in the main text): 2 (p = .011), 5 (p = .002), 6 (p < .001), 8 (p = .011), 11 (p = .003), 12 (including the two follow-up interaction analyses) (ps < .010), 14 (p = .012), 17 (including the follow-up interaction analysis in men only) (ps = .001), 18 (including the two follow-up interaction analyses) (ps < .009). Age was significantly negatively associated with the outcome variable in the following regressions (see Table 1 in the main text): 5 (p = .011), 6 (p = .003), 11 (p = .008), and 12 (including the follow-up interaction analysis in individuals assigned female at birth) (ps < .004). All other instances, age and experimenter were not significant (ps > .063).

Table S13. Mean and standard deviation for the anthropometric variables included in allometry analyses, by group.

		Het men	Het women	Individuals assigned male at birth (AMAB)	Individuals assigned female at birth (AFAB)
Average 2D:4D	*M*	0.96	0.98	0.97	0.98
	SD	0.03	0.03	0.03	0.03
	n	279	281	641	679
Left 2D:4D	*M*	0.96	0.97	0.97	0.97
	SD	0.03	0.03	0.03	0.03
	n	278	280	640	678
Right 2D:4D	*M*	0.96	0.98	0.97	0.98
	SD	0.03	0.03	0.03	0.03
	n	278	280	639	678
Average finger length	*M*	72.96	67.16	72.57	67.30
	SD	3.68	3.81	3.72	3.89
	n	279	281	641	679
Average left finger length	*M*	73.12	67.10	72.67	67.30
	SD	3.76	3.85	3.81	3.94
	n	278	281	640	679
Average right finger length	*M*	72.80	67.22	72.46	67.29
	SD	3.77	3.87	3.77	3.95
	n	279	281	641	679
Average hand length	*M*	185.23	169.32	184.61	169.83
	SD	7.58	7.55	7.88	7.77
	n	279	281	641	678
Left hand length	*M*	18.57	16.95	18.51	17.01
	SD	0.76	0.76	0.80	0.79
	n	279	281	641	678
Right hand length	*M*	18.48	16.91	18.42	16.95
	SD	0.78	0.77	0.81	0.79
	n	278	281	640	678
Height	*M*	170.22	156.77	169.83	157.31
	SD	5.97	5.86	5.97	5.54
	n	279	279	626	700

Note. Het = heterosexual. Height and hand length were measured in centimetres (cm) and finger length was measured in millimetres (mm). AMAB includes heterosexual men, gay men, and *sao praphet song*. AFAB includes heterosexual women, *dees*, bisexual women, lesbian women, and *toms*. Values were calculated in SPSS.

Full Regression Results

Tables S14, S15, and S16 provide results from all non-redundant pairwise comparisons from the regressions presented in the main text. All analyses statistically controlled for age and experimenter, but these results are not shown in tables and are instead outlined next. Experimenter was significantly negatively associated (i.e., larger measurement values were associated with DPV) with arm length for all comparisons (*p*s < .001), with weight for comparisons within the individuals assigned male at birth (AMAB) (*p* = .025), and for left 2D:4D for comparisons within the individuals assigned female at birth (AFAB) (*p* = .016). Experimenter was significantly positively associated (i.e., larger measurement values were associated with LAC) with right 2D:4D (*p*s < .032), left-hand width-to-length ratio (*p*s < .001), and right-hand width-to-length ratio (*p*s < .001) for all comparisons. Age was significantly negatively associated (i.e., being older was associated with smaller measurement values) with arm length (*p*s < .001), height (*p*s < .001), and leg length (*p*s < .001) for all comparisons, and with right 2D:4D for comparisons within AFABs (*p* = .002) and within heterosexual participants (*p* = .005). Age was significantly positively associated (i.e., being older was associated with larger measurement values) with weight for comparisons within AFABs (*p* = .003), and with left-hand width-to-length ratio (*p*s < .001) and right-hand width-to-length ratio for all comparisons (*p*s < .001). In all other instances, experimenter and age were not significant (*p*s > .085).

Table S14. Results of regressions with each biomarker as the dependent variable and heterosexual men versus heterosexual women as the independent variable.

	n	*β*	*p*	Cohen's *d*	95% confidence interval	
Weight	553	**-.138**	**< .001**	-0.14	-0.31	0.03
Height	554	**-.209**	**< .001**	-0.21	-0.38	-0.04
Arm Length	531	**-.171**	**< .001**	-0.17	-0.34	0.00
Leg Length	532	**-.190**	**< .001**	-0.19	-0.36	-0.02
Left 2D:4D	554	**.056**	**< .001**	0.06	-0.11	0.22
Right 2D:4D	554	**.054**	**< .001**	0.05	-0.11	0.22
Left HWLR	555	**-.058**	**< .001**	-0.06	-0.22	0.11
Right HWLR	554	**-.068**	**< .001**	-0.07	-0.23	0.10

Note. HWLR = hand width-to-length ratio. Age and experimenter were statistically controlled in all analyses (results not shown). Heterosexual men are coded 0 and heterosexual women are coded 1. Analyses were conducted in Mplus.

Table S15. Results of regressions with each biomarker as the dependent variable and dummy coded group as independent variables within individuals assigned female at birth.

Coded 0 →		Heterosexual Women Versus…				*Toms* Versus…			Lesbian Women Versus…		Bisexual Women Versus…
Coded 1 →		*Toms*	Lesbian Women	Bisexual Women	*Dees*	Lesbian Women	Bisexual Women	*Dees*	Bisexual Women	*Dees*	*Dees*
Weight	*β*	**.332**	-.018	.009	-.060	**-.350**	*-.323*	**-.392**	.027	-.042	-.069
	p	**.002**	.864	.955	.585	**.003**	*.053*	**.002**	.872	.740	.686
Height	*β*	.153	.133	-.029	-.099	-.020	-.181	**-.252**[a]	-.162	-.232	-.070
	p	.134	.295	.842	.385	.877	.222	**.028**[a]	.336	.100	.657
Arm	*β*	-.148	.047	-.151	-.118	.195	-.003	.030	-.198	-.165	.033
Length	*p*	.168	.728	.345	.315	.162	.986	.795	.287	.271	.848
Leg	*β*	.130	*.249*	-.020	-.149	.119	-.150	**-.279**	-.269	**-.398**	-.129
Length	*p*	.225	*.056*	.885	.224	.367	.298	**.026**	.100	**.008**	.414
Left	*β*	*.206*	-.102	.019	-.017	**-.308**	-.187	*-.223*	.120	.085	-.035
2D:4D	*p*	*.056*	.461	.908	.891	**.032**	.268	*.069*	.530	.587	.843
Right	*β*	.103	-.050	.012	-.064	-.153	-.091	-.166	.062	-.014	-.075
2D:4D	*p*	.370	.733	.940	.568	.310	.576	.151	.742	.929	.641
Left	*β*	.037	-.204	.032	.002	-.241	-.005	-.035	.235	.206	-.030
HWLR	*p*	.740	.146	.854	.983	.101	.976	.768	.246	.163	.870
Right	*β*	**.213**[a]	-.012	-.003	**.299**	-.225	-.216	.086	.009	**.311**[a]	.301
HWLR	*p*	**.049**[a]	.936	.988	**.004**	.155	.225	.461	.964	**.048**[a]	.088

Note. Sample sizes are: 693 (weight), 695 (height), 653 (arm length and leg length), 673 (left and right 2D:4D), 669 (left and right hand width-to-length ratio, HWLR). Age and experimenter were statistically controlled in all analyses (results not shown). [a]These comparisons are no longer significant after controlling for education and are not interpreted in the main results. Analyses were conducted in Mplus.

Table S16. Results of regressions with each biomarker as the dependent variable and dummy coded group as independent variables within individuals assigned male at birth.

Coded 0 →		Heterosexual Men Versus…		*Sao Praphet Song* Versus…
Coded 1 →		Gay Men	*Sao Praphet Song*	Gay Men
Weight	*β*	**-.195**	*-.222*	.027
	p	**.041**	*.051*	.813
Height	*β*	-.117	**-.276**	.159
	p	.227	**.005**	.133
Arm	*β*	-.157	**-.227**	.071
Length	*p*	.092	**.027**	.512
Leg	*β*	-.133	**-.299**	.166
Length	*p*	.175	**.004**	.119
Left	*β*	**.235**	**.242**	-.007
2D:4D	*p*	**.020**	**.010**	.944
Right	*β*	.097	.073	.025
2D:4D	*p*	.308	.460	.819
Left	*β*	*-.182*	-.141	-.041
HWLR	*p*	*.053*	.156	.702
Right	*β*	-.131	-.068	-.062
HWLR	*p*	.169	.473	.550

Note. Sample sizes are: 623 (weight), 622 (height), 602 (arm length), 607 (leg length), 634 (left 2D:4D), 633 (right 2D:4D), 635 (left hand width-to-length ratio, HWLR), and 634 (right HWLR). Age and experimenter were statistically controlled in all analyses (results not shown). Analyses were conducted in Mplus.

References

1. Skorska, M. N., Coome, L. A., Peragine, D. E., Aitken, M., & VanderLaan, D. P. Data from: An anthropometric study of sexual orientation and gender identity in Thailand. Scholar's Portal Dataverse (2021). Doi:10.5683/SP2/XV14U3
2. Gooren, L. J., Sungkaew, T., Giltay, E. J., & Guadamuz, T. E. Cross-sex hormone use, functional health and mental well-being among transgender men (*Toms*) and transgender women (*Kathoeys*) in Thailand. *Cult. Health Sex.* **17**, 92–103 (2015). Doi:10.1080/13691058.2014.950982
3. Winter, S. Thai transgenders in focus: Demographics, transitions and identities. *Int. J. Transgend.* **9**, 15–27 (2006). Doi:10.1300/J485v09n01
4. Galis, F., Ten Broek, C. M. A., Van Dongen, S., & Wijnaendts, L. C. D. Sexual dimorphism in the prenatal digit ratio (2D:4D). *Arch. Sex. Behav.* **39**, 57–62 (2010). Doi:10.1007/s10508-009-9485-7
5. Skorska, M. N. & Bogaert, A. F. Pubertal stress and nutrition and their association with sexual orientation and height in the Add Health data. *Arch. Sex. Behav.* **46**, 217–236 (2017). Doi:10.1007/s10508-016-0800-9
